# Malnutrition-Inflammation Score VS Phase Angle in the Era of GLIM Criteria: A Cross-Sectional Study among Hemodialysis Patients in UAE

**DOI:** 10.3390/nu11112771

**Published:** 2019-11-14

**Authors:** Mirey Karavetian, Nada Salhab, Rana Rizk, Kalliopi Anna Poulia

**Affiliations:** 1 Department of Health Sciences, College of Natural Health Sciences, Zayed University, Dubai P.O. Box 19282, UAE; Mirey.Karavetian@zu.ac.ae; 2 School of Nutrition and Translational Research in Metabolism, Faculty of Health Medicine and Life Sciences, Maastricht University, 6200 MD Maastricht, The Netherlands; 3 Institut National de Santé Publique, d’Épidémiologie Clinique et de Toxicologie, The Lebanese University, Fanar 90-1965, Lebanon; r.rizk@maastrichtuniversity.nl; 4 School of Public Health and Primary Care, Faculty of Health Medicine and Life Sciences, 6200 MD Maastricht, The Netherlands; 5 Department of Nutrition and Dietetics, Laiko General Hospital of Athens, 11527 Athens, Greece

**Keywords:** malnutrition, malnutrition-inflammation score, phase angle, Global Leadership Initiative on Malnutrition

## Abstract

(1) Background: Malnutrition is prevalent in hemodialysis (HD) patients and is associated with an increased risk of morbidity and mortality. The aim of this study was to explore the prevalence of malnutrition using the malnutrition-inflammation score (MIS) and phase angle (PhA) and compare their concordance with the new Global Leadership Initiative on Malnutrition (GLIM) criteria for the diagnosis of malnutrition. (2) Methods: Seventy HD patients were assessed. Malnutrition was diagnosed based on the GLIM criteria and MIS questionnaire. The agreement between the diagnostic tools (MIS, PhA derived from the bioelectrical impedance analysis (BIA), and GLIM criteria) was assessed. The optimal gender-specific cutoff points were identified for the PhA according to the GLIM criteria. (3) Results: Almost half of the sample was diagnosed as malnourished according to the MIS (48.57%) and GLIM criteria (54.29%). A fair agreement was observed between the GLIM criteria, MIS (k = 0.202), and PhA (k = 0.279) among the malnourished patients. The PhA had better sensitivity but worse specificity compared to the MIS. The optimum cutoff points of PhA to detect malnutrition according to the GLIM criteria were a PhA value of ≤5.7° for males and ≤3.8° for females. (4) Conclusion: The MIS performed slightly better than PhA in the diagnosis of malnutrition among HD patients within the spectrum of the GLIM criteria.

## 1. Introduction

Malnutrition in hemodialysis (HD) is a well-established condition and often co-exists with inflammation [1]. Between 50% to 75% of HD patients show signs of the malnutrition-inflammation complex syndrome (MICS) depending on the diagnostic tool used [2]. This syndrome is closely associated with adverse patient outcomes such as frailty, depression, higher morbidity and mortality, and worsened quality of life (QoL) [2,3]. The global outcome is a vicious cycle of MICS and its consequences, leading to chronicity [2]. Accordingly, a careful and periodic nutritional screening, coupled with a timely diagnosis and the close monitoring of nutritional status, are cornerstones for implementing tailored interventions for the prevention and treatment of MICS [2]. This necessitates the early identification of nutritional risk with tools that are accurate and sensitive [4,5].

Traditionally, the tools employed to detect MICS in HD patients ranged from anthropometry, to biochemical parameters, and numerous composite scores parallel to clinical judgment [6,7,8]. Among the latter, the malnutrition-inflammation score (MIS) emerged as a comprehensive quantitative scoring system for diagnosing MICS [1,9,10,11]. However, its use in clinical practice requires an interview with the patient, a thorough physical examination of subcutaneous body fat and signs of muscle wasting, in addition to biochemical variables including serum albumin level, total iron-binding capacity (TIBC), or transferrin level [9]. Within the time constraints reported among renal dietitians [12], there is a pressing need for an accurate, easy-to-use, and cost-effective diagnostic tool. In recent years, bioelectrical impedance analysis (BIA)-derived phase angle (PhA) has gained attention in this regard, especially since the Academy of Nutrition and Dietetics stated that the use of BIA is appropriate in chronic kidney disease (CKD) patients [13]. PhA is proportional to the ratio of reactance and resistance between body cell mass and fat-free mass [14], and is assumed to indicate cell integrity [15]. In various clinical fields, PhA was shown to be a reliable marker for the detection of early malnutrition [16,17]. 

The primary aim of this study was to explore the prevalence of malnutrition in a sample of HD patients in a hospital-based dialysis unit in the United Arab Emirates (UAE) using the MIS and BIA, and compare them with the new Global Leadership Initiative on Malnutrition (GLIM) criteria for the diagnosis of malnutrition. The secondary aim was to find out the gender-specific cutoff point of PhA for the studied population.

## 2. Materials and Methods 

### 2.1. Study Design

A cross-sectional study was conducted in September 2017 in a randomly chosen tertiary hospital-based HD unit in the UAE. The study protocol was designed in accordance with the Helsinki Ethical Declaration. The study was registered at ClinicalTrials.gov (ID: NCT03131804). The study belongs to a larger clinical trial for which the trial protocol was previously published [18]. Ethical approval was obtained from Zayed University and the Ministry of Health and Prevention (MOHP). All eligible and consenting patients receiving HD in the unit were recruited to the study. Due to the lack of official renal registry in the UAE, we were unable to obtain the total number of HD patients. Thus, a power analysis could not be carried out. The eligible candidates were stable adult patients on HD, free of acute diseases (cancer, active infections, myocardial infarction, and acute pulmonary diseases), who had not been hospitalized in the past 6 months, and were willing to participate in the study. Patients who were fitted with a heart pacemaker, pregnant, or did not meet the inclusion criteria were excluded. All patients who consented also completed the study. All demographic and clinical data were collected from the electronic medical records. Initially, the original trial was planned to be a multicenter trial. However, we obtained ethical approval to only one center excluding patients with hepatopathy. Thus, the study was performed in one center. 

### 2.2. Malnutrition-Inflammation Score (MIS)

The MIS is a validated tool frequently used for assessing the malnutrition-inflammation status of patients. This is a composite score of 10 components, each with four levels of severity ranging from 0 (normal) to 3 (severely abnormal). The scoring sheet is composed of four sections: (1) patients’ related medical history including weight change in edema-free post-HD body weight in the past 6 months, dietary intake, gastrointestinal symptoms, functional capacity, and comorbidities; (2) physical exam of subcutaneous body fat and signs of muscle wasting according to the Subjective Global Assessment (SGA) criteria; (3) body mass index (BMI); and (4) laboratory parameters, i.e., serum albumin level and TIBC [9]. The sum of all components ranges from 0 (normal) to 30 (severely abnormal). A higher score reflects a more severe degree of malnutrition and inflammation. The assessment was performed by trained dietitians at the end of the HD session. To avoid bias in the collection of data, the dietitians who conducted the MIS were different from those who conducted the BIA.

### 2.3. Malnutrition Using the Global Leadership Initiative on Malnutrition (GLIM) Criteria 

In 2018, the Global Leadership Initiative on Malnutrition (GLIM) criteria were introduced [19]. GLIM is a two-step approach for malnutrition diagnosis. The first step incorporates initial screening with any validated screening tool in order to identify “at risk” patients. The second step is the assessment for the diagnosis and grading of the severity of malnutrition. The assessment criteria for malnutrition include the phenotypic criteria: (1) non-volitional weight loss (weight loss of > 5% within the past 6 months); (2) low BMI (< 20 kg/m^2^ if < 70 years and < 22 kg/m^2^ if ≥ 70 years); (3) reduced muscle mass (fat-free mass index (FFMI)of < 17 kg/m^2^ for males and < 15 kg/m^2^ for females) [20]; and (4) two etiologic criteria consisting of reduced food intake and inflammation or disease burden. To diagnose malnutrition, at least one phenotypic and one etiologic criterion should be present. For this study, we assessed the non-volitional weight loss based on the previous European Society for Parenteral and Enteral Nutrition (ESPEN) recommendation, which is >5% within the last 3 months, because these were the published recommendations at data collection point [20]. Furthermore, the phenotypic grading of malnutrition was described as Stage 1 (moderate) and Stage 2 (severe) malnutrition [19] using the sarcopenia cutoff points for low muscle quantity, where patients with low appendicular muscle mass (ASM) (< 20 kg in males and < 15 kg in females) were classified as Stage 2 malnourished patients. Higher or equal ASM values were indicated as Stage 1 malnutrition. ASM was derived from the skeletal muscle mass (SMM) measured by the BIA [21], and the cutoff points were taken from the consensus of the European Working Group on Sarcopenia in Older People 2 (EWGSOP2) [21]. Furthermore, fat mass index (FMI) was measured independently of GLIM for the sake of finding potential patients with high adiposity coupled with low muscle mass. Different studies reported a range of cutoff points, the highest numbers being FMI ≥7 kg/m^2^ for males and 9.5 kg/m^2^ for females, as indicative of metabolic syndrome [22,23,24].

### 2.4. Phase Angle (PhA)

The reaction of the body to an electric current when using a BIA comprises two components: (1) resistance, which is the restriction to the electrical current flow due to extracellular water (ECW) and intracellular water (ICW); and (2) reactance, which is the resistive effect of the body tissues and cell membranes. PhA is a parameter that reflects the amount of reactance in the human body relative to the amount of resistance. PhA is calculated based on the following formula: reactance/resistance*180/π [14]. PhA decreases with increased inflammation/disease severity [25] and age [26]. In the current study, BIA was performed using the SECA (mBCA 525-Germany) device in a supine position according to the ESPEN guidelines on BIA application [27]. The device analyzed body composition through 8 points of contact where two electrodes were connected to each of the right and left arm and leg. Patients with a voided bladder were assessed within 10 minutes after the HD session according to the manufacturer’s recommendations. The standardized PhA value, adjusted for the age and gender of the patient, was directly generated by the BIA. Height was measured in the clinic during the session and integrated in the data of the BIA machine.

### 2.5. Statistical Analysis

The collected data were analyzed using IBM SPSS Statistics 21.0 package. Descriptive analysis was used to summarize the study variables and to check for out-of-range values. Kolmogorov-Smirnov tests were used to assess data normality. The categorical variables were described using frequencies and percentages, while means and standard deviations were used to represent the normally distributed continuous variables. The medians and interquartile range (IQR) values were used for skewed data. Pearson’s correlation coefficient was used to examine the relationship between the MIS and PhA. The concordance of the MIS and PhA were analyzed using the MedCalc software. Further analysis was done to detect which of these assessors predicted malnutrition best based on the GLIM criteria. Thus, the area under the curve (AUC) based on receiver operating characteristic (ROC) statistics [28] and the positive and negative likelihood ratios (LR+ and LR− respectively) were calculated. An area under the ROC curve equal to 0.5 indicates that a tool could not distinguish between the two groups. When equal to 1, it indicates perfect separation of the values of the two groups. Furthermore, sensitivity, specificity, positive and negative predictive values, and likelihood ratios were calculated. Two-tailed *p* ≤ 0.05 was reported for all statistical tests. To assess the agreement between the old and new malnutrition assessors, Cohen’s kappa (κ) was used to compare (1) a newly established malnutrition criteria (the GLIM criteria) with an already established tool (MIS); and (2) the GLIM criteria with a newly emerging criteria (PhA) with κ ranges from −1 to +1. Based on the guidelines from Altman, a κ < 0.2 represents a poor strength of agreement, and 0.21 < κ < 0.40, 0.41 < κ < 0.60, 0.61 < κ < 0.80, and 0.81 < κ < 1.00 represent a fair, moderate, good, and very good agreement respectively. Finally, the positive predictive value (PPV), negative predictive value (NPV), and the likelihood ratios were used to study the concordance of the GLIM criteria with the MIS (>10) and PhA (≤5.7°). The latter was identified in this study as the cutoff point for the general sample. Below this, the PhA indicated malnutrition. These measures were included as they are important in public health and health planning [29].

## 3. Results

### 3.1. Patients’ Characteristics

Seventy adult HD patients were included in the study. The mean age was 54.61 ± 12.79 years and the mean BMI was 27.22 ± 6.48 kg/m^2^. Hypertension was the most common comorbidity (88.57%), followed by diabetes (65.71%) and cardiovascular diseases (40.00%). The descriptive data of the sample are presented in Table 1.

The mean MIS of the participants was 9.40 ± 3.07 and SMM was within normal values, however the FFMI indicated low muscle mass and borderline malnourishment (males: 15.08 ± 2.74; and females 17.90 ± 3.44). Fat mass index was indicative of excess fat and only 2.9% of the sample had low FMI. Finally, the mean of the BIA-derived PhA was 4.66 ± 1.21°. The latter was analyzed for 69 patients due to one item of missing patient data.

Almost half of the sample was identified to be malnourished with the MIS (48.57%) and GLIM criteria (54.29%). Further analysis using the GLIM criteria staging showed that almost 70% of the malnourished were actually severely malnourished.

### 3.2. Concordance of GLIM with MIS and PhA

**Figure 1:**
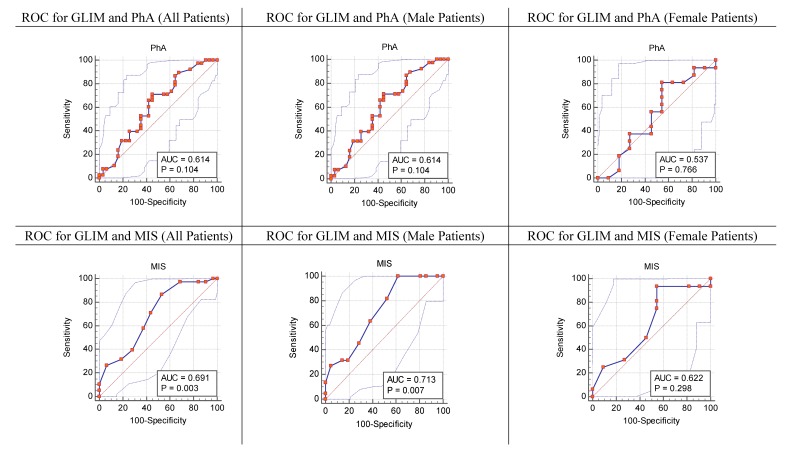
Receiver operating characteristic (ROC) curve for phase angle (PhA) and the malnutrition-inflammation score (MIS) according to the Global Leadership Initiative on Malnutrition (GLIM) criteria.

The area under the curve based on ROC curve statistics was presented in Figure 1. The identified cutoff points of PhA according to the GLIM criteria for the whole sample was PhA ≤5.7° (males: ≤ 5.7°; females: ≤3.8°). As for the MIS, the cutoff point of MIS > 10, retrieved from the literature, was used [9]. 

The MIS seemed to be slightly better correlated with the GLIM criteria according to the AUC compared with the PhA (AUC_MIS_ = 0.691, confidence interval = 0.569–0.796, *p* = 0.003, vs. AUC_PhA_ = 0.614, confidence interval = 0.489–0.729, *p* = 0.104) (Figure 1). PhA had a better sensitivity (86.84%) compared with the MIS (39.47%), but a worse specificity (35.48 vs. 71.87 respectively). The MIS was found to have better LR+ and LR at the same time. 

### 3.3. Strength of Agreement between the GLIM Criteria vs. the MIS, and the GLIM Criteria vs. PhA 

The strength of agreement between the MIS and GLIM (Table 2) criteria in diagnosing malnutrition according to Cohen’s kappa was fair and not statistically significant (κ = 0.202; *p* = 0.089). The strength of agreement between the PhA and GLIM criteria in diagnosing malnutrition according to Cohen’s kappa was fair and statistically significant (κ = 0.234; *p* = 0.029).

## 4. Discussion

To our best knowledge, this is the first study that has identified the prevalence of malnutrition among a selected group of HD patients in the UAE while assessing the concordance of the BIA-derived PhA (a holistic assessment tool) and MIS (an established assessment tool) with the GLIM diagnostic criteria for malnutrition. 

This study identified almost half of the HD patients as malnourished using the MIS and showed a concordance between the two assessors. Alarmingly, most of these patients were also severely malnourished. 

There was a fair agreement for both assessors with the GLIM criteria and it was statistically significant for PhA but not MIS. The reasons for the lack of stronger agreement can be attributed to the small sample size. Moreover, the lack of strong agreement between the MIS and GLIM criteria may be due to the fact that GLIM assessed muscle mass using objective measures whereas the MIS used subjective ones. Since PhA is BIA-derived and fully objective, we conducted a further analysis to identify the specific cutoff points for PhA of malnutrition in the general population. The angle at 5.7° and below showed to be the indicator for malnutrition. The specificity and sensitivity of PhA to GLIM was seen to be optimal at 5.7° for males and 3.8° for females. These numbers might be considered as possible cutoff points to screen HD patients for malnutrition. In another setting, different cutoff points for PhA appeared to be a useful in screening nutritional status among males (5.0°) and females (4.6°) [16]. Moreover, our results were in line with the findings of Bansal [30], where they demonstrated a PhA of <5.59° among chronic kidney disease patients to be associated with mortality.

Based on our analysis, PhA had higher sensitivity than the MIS using the GLIM criteria as a reference standard. This means that PhA can lead to a greater number of diagnosis of malnourished cases when compared to the MIS. In fact, metabolic changes in cell membranes are first affected by malnutrition [31]. As PhA reflects on cell integrity, it could detect malnutrition at an early stage. Unfortunately, when the malnutrition-inflammation syndrome complex (MISC) develops in the patient, it forms a vicious cycle that is difficult to treat; thus, measuring the PhA may be a tool to prevent MISC from being established.

In addition, the AUC suggested that, clinically, the MIS achieved better performance than PhA in identifying malnutrition in HD patients since it was more sensitive, and the AUC curve with the GLIM criteria resulted in statistically significant results. It must be noted that the difference between the AUC of the MIS and PhA with the GLIM criteria was <0.08, denoting almost similar diagnostic capabilities when it came to the whole sample. With the current results, we concluded that the MIS should be used as a better diagnostic tool than PhA at this stage. This was expected since the MIS is a tool especially designed for HD patients. In addition, the superiority of the MIS over the PhA might be related to the small sample size we had. In fact, a recent review reported that PhA may not be an accurate indicator of malnutrition [32]. However, PhA is a very new indicator and further research is needed for its accuracy, appropriateness, and cutoff points, particularly in HD patients.

The literature is clear on the relationship of malnutrition and low muscle mass with higher mortality [33]. Thus, our results may be used in interventions like improved protein intake and physical exercise in HD patients to prolong longevity and increase QoL.

There were a few limitations to this study, such as the sample not being powerful enough to detect the prevalence of malnutrition in the UAE and the use of a convenience selection sampling technique. These limitations did not allow us to generalize our findings to the whole HD population in the UAE. Finally, the assessments were based on a single measurement and this might have influenced the results, knowing the nature of CKD and the health fluctuations that affect HD patients. 

The overall results of this study can lead us to speculate that the clinical difference between PhA and the MIS are not very far apart, and that both have their strengths and weaknesses. However, they do not result in major differences for the clinician and the patient. Consequently, the current research team invites future studies to look into larger samples for this patient subgroup and to validate the clinical effectiveness of using PhA as a practical tool for malnutrition screening.

## 5. Conclusions

Malnutrition is widespread in the HD population in the UAE. Clinically, in the current sample, the MIS performed slightly better than PhA in the diagnosis of malnutrition when using the GLIM criteria as a reference, but both tools may perform equally on a large sample. Prioritizing malnutrition screening in this population and integrating cost-effective, sensitive, and specific tools within routine practice is advocated as a result of the findings of this study. 

## Figures and Tables

**Table 1:** Demographic and characteristics of study participants (*n* = 70).

	**Total *n* = 70**
	**Mean (SD)**
Age (years)	54.61 (12.79)
BMI (kg/m^2^)	27.22 (6.48)
PhA (°)	4.66 (1.21)
MIS	9.40 (3.07)
FMI (kg/m^2^)	10.15 (5.00)
SMM (kg)	19.67 (6.44)
Fat (kg)	26.70 (12.57)
	**Median (IQR)**
FFMI (kg/m^2^)	17.09 (3.33)
FFM (kg)	42.92 (15.14)
TBW (L)	32.15 (11.4)
ECW (L)	15.20 (4.7)
	**N (%)**
Gender: Male	43 (61.4)
Comorbidities * Diabetes Hypertension Cardiovascular Diseases Others	46 (65.71) 62 (88.57) 28 (40.00) 37 (52.86)
Dialysis Vintage <1 year 1–4 years >4 years	5 (7.14) 35 (50.00) 30 (42.86)
Malnourished as per GLIM Criteria	38 (54.29)
Stage 1: Moderate Malnutrition	12 (31.58)
Stage 2: Severe Malnutrition	26 (68.42)
Malnourished as per the MIS (>10)	34 (48.57)

BMI: body mass index; PhA: phase angle; MIS: malnutrition-inflammation score; FMI: fat mass index; SMM: skeletal muscle mass; FFMI: fat-free mass index; TBW: total body water; ECW: extracellular water; GLIM: Global Leadership Initiative on Malnutrition; SD: standard deviation; IQR: interquartile range. * Percentages do not sum up due to aggregated error from multiple answers. PhA was analyzed for 69 patients. Cutoff points for malnutrition: MIS (>10); SMM (males ˂ 0 kg; females ˂ 15 kg); FMI (males < 1.2 kg/m^2^; females < 3.8 kg/m^2^); FFMI (males < 17 kg; females < 15 kg).

**Table 2:** Concordance of the GLIM criteria with the MIS (>10) and PhA (≤5.7°).

Criterion	MIS > 10	PhA ≤ 5.7°
Sensitivity (%)	39.47	86.84
Specificity (%)	71.87	35.48
Positive predictive value (%)	50.0	62.63
Negative predictive value (%)	62.5	68.68
Positive likelihood ratio (LR+)	1.40	1.35
Negative likelihood ratio (LR−)	0.84	0.37
K value (*p*)	0.202 (0.089)	0.234 (0.029)
Area under the curve (AUC)	0.691	0.614
